# Road Traffic Injury Characteristics, Severity, and Management Outcome among Victims Treated at the Emergency Department of Jimma Medical Center, Jimma, Ethiopia, 2024

**DOI:** 10.4314/ejhs.v35i3.8

**Published:** 2025-05

**Authors:** Demuma Amdisa, Netsanet Workneh, Leta Alemu, Getachew Tilahun, Nega Jibat, Shemsedin Amme

**Affiliations:** 1 Department of Health Behavior and Society, Faculty of Public Health, Institute of Health, Jimma University; 2 WHO Trauma Registry, Jimma Medical Center; 3 Department of Media and Communication Studies, College of Social Sciences and Humanities; 4 Department of Sociology, College of Social Sciences and Humanities; 5 Department of Nursing, Faculty of Health Sciences

**Keywords:** Road traffic, injury, victims, accident, trauma care, Ethiopia

## Abstract

**Background:**

Road traffic injuries (RTIs) are a significant public health and development challenge. This study assessed injury characteristics, severity, and management outcomes of RTI victims at Jimma Medical Center, Jimma, Ethiopia.

**Method:**

A retrospective chart review was conducted on RTI victims treated at the Emergency Department of Jimma Medical Center between September 2021 and June 2022. The World Health Organization's Road traffic injury surveillance tool and the Kampala Trauma Score II (KTS II) were used to evaluate injury severity. Data were entered using EpiData version 3.4 and analyzed with SPSS version 20. Descriptive statistics and cross-tabulations were used in the analysis.

**Results:**

Of the 391 victims included, 270 (69.1%) were male, with a median age of 25 years. Pedestrians (52.9%, n=207) were the most affected group, followed by drivers (17.4%, n=68). Cars accounted for over half of the accidents (52.2%, n=204), while motorcycles were involved in 31.7% (n=124). Musculoskeletal (35.5%) and head injuries (25.6%) were the most common. Severe injuries were reported in 62.2% (n=140) of cases. Fewer than half (44.5%, n=174) of the victims were transported by ambulance, and only 21% (n=82) received first aid from healthcare providers. Most victims (93.5%, n=359) survived, while 6.4% (n=25) died.

**Conclusion:**

RTIs present a considerable burden in this setting with pedestrians mostly affected. Targeted interventions should address road safety, enhance pre-hospital care, and improve medical documentation. A multi-sectoral injury surveillance system and identification of key risk factors are essential for reducing RTI impacts.

## Introduction

Road traffic deaths have steadily increased from 1.15 million in 2000 to 1.35 million in 2018 (1). This burden falls disproportionately on low- and middle-income countries (LMICs), while high-income countries have seen declines (2). Africa reports the highest RTI mortality rate globally, and projections suggest RTIs will soon become the leading cause of death among children aged 5–15 years (3). In East Africa, Ethiopia ranks second to Kenya in reported road traffic fatalities (4). RTIs significantly affect socio-economic development, causing economic losses of up to 5% of GDP in LMICs (5). Beyond fatalities, non-fatal RTIs result in long-term disability, psychological trauma, and reduced quality of life (6).

RTIs stem from numerous factors, including risky driving behaviors, pedestrian demographics, vehicle condition, infrastructure, and environmental factors (7,8,9). Despite these challenges, RTIs are preventable, and global initiatives to enhance road safety have gained momentum (1). The Sustainable Development Goals (SDGs) include a target to halve global road traffic deaths and injuries by 2030 (10).

Localized data is critical for effective prevention strategies. While a previous study by Mamo et al. (11) examined RTIs in Jimma, it did not assess injury severity. This study aims to fill that gap by analyzing injury characteristics, severity, and management outcomes among RTI victims treated at Jimma Medical Center's Emergency Department.

## Methods and Materials

**Study setting**: The study was conducted at Jimma Medical Center (IMC), in Jimma City, Ethiopia, 352 km southwest of Addis Ababa. JMC is the only teaching and referral hospital in southwestern Ethiopia, serving approximately 15 million people. It provides services to about 15,000 inpatients, 160,000 outpatients, 11,000 emergency cases, and 4,500 deliveries annually. The Emergency Department (ED) alone manages over 102,000 patients yearly, including 1,800 trauma cases.

**Study design and period**: A retrospective chart review was conducted for RTI victims treated between September 2021 and June 2022.

**Study population**: All RTI victims treated at the Emergency Department during the study period were eligible. Cases with incomplete data (missing demographics, injury details, treatment, or outcomes) or who were dead on arrival were excluded.

**Variables**: The study variables included demographic characteristics (age, sex, residence), injury characteristics, injury severity, pre-hospital and hospital care, and management outcomes.

**Data collection**: Data were extracted using the WHO road traffic injury surveillance tool. Victims were followed until discharge or death from the Emergency Department. Injury severity was assessed using the Kampala Trauma Score II (KTS II), as previously applied in similar studies (10). Data were collected by five trained health researchers. KTS II scoring details are presented in Table 1.

**Table 1:** Description of Kampala Trauma Score (KTS II)

Category and Description		Score
A.	Age (in Years)	
	5-55	1
	<5 or >5	0
B.	Systolic Blood Pressure on admission	
	More than 89 mm Hg	2
	Between 89-50 mm Hg	1
	Equal or below 49 mm Hg	0
C.	Respiratory rate on admission	
	0-29/minute	2
	30+	1
	≤9/minutes	0
D.	Neurological Status	
	Alert	3
	Responds to Verbal stimuli	2
	Responds to painful stimuli	1
	Unresponsive	0
E.	Score for a series of injuries	
	None	2
	One injury	1
	More than one	0

Kampala Trauma Score total = A + B + C + D + E; Scores, 9–10: Mild injury; 7–8: Moderate injury; 6 or less (≤6): Severe injury [10]

**Data processing and analysis**: Data were cleaned, coded, and entered into EpiData version 3.1 and analyzed with SPSS version 22. Descriptive statistics, frequency distributions, cross-tabulations, and graphs were used to present the results.

**Ethical considerations**: Ethical approval was obtained from the Institutional Review Board of Jimma University, Institute of Health. Patient confidentiality was maintained, and personal identifiers were not recorded.

## Results

**Socio-demographic characteristics**: Of 401 cases, 391 were included after excluding 10 for incomplete data. Most victims were male (69.1%, n=270), with a median age of 25. Individuals under 20 years represented the largest age group (37.3%, n=146) (Table 2).

**Table 2:** Socio-demographic Characteristics of Victims of Road Traffic Accident, September-June 2021/22, at Jimma Medical Center, Jimma, Ethiopia, 2022

Socio-Demographic Characteristics	Frequency (%)
Sex	
Male	270 (69.1)
Female	121 (30.9)
Age Category	
< 20 Years	146 (37.3)
20-29 Years	76 (19.4)
30-39 Years	67 (17.1)
40-49 Years	46 (11.8)
50-59 Years	25 (6.4)
>60 years	31 (7.9)
Residence	
Urban	278 (71.1)
Rural	109 (27.9)

**Injury characteristics and severity**: Cars were involved in 52.2% (n=204) of incidents, followed by motorcycles (31.7%, n=124). Pedestrians were the most affected (52.9%, n=207), followed by drivers (17.4%, n=68). The majority of incidents occurred in the late afternoon (34.3%, n=107), although arrival times were undocumented for 20% of cases.

Musculoskeletal (35.5%) and head injuries (25.6%) were most common, with 22.5% sustaining multi-region trauma. Single injuries occurred in 62.1% (n=243), multiple injuries in 34.5% (n=135), and 3.3% (n=13) were undocumented (Table 3).

**Table 3:** Site of Injuries among the Road Traffic Accident Victims at Emergency Department of Jimma Medical Center, Jimma, Ethiopia, September—June 2021/22

Site of Injury	Frequency (%)
Musculoskeletal	139 (35.5)
Head	100 (25.6)
Head and Musculoskeletal	39 (10)
Chest	13 (3.3)
Pelvis	7 (1.8)
Abdominal	5 (1.3)
Multiple injuries	88 (22.5)

Based on KTS II, 62.2% (n=140) of victims had severe injuries, 33.3% (n=75) moderate, and 4.4% (n=10) mild. Mortality was 0% for mild injuries, 12.5% (n=1) for moderate, and 87.5% (n=7) for severe injuries.

**Post-crash management and outcome**: Only 21% (n=82) received first aid from healthcare providers, while 45.8% (n=179) received no first aid. Layperson first aid was recorded for 11.5% (n=45), and 21.7% (n=85) had no documentation. Most victims (58.8%, n=230) presented without referral, and 39.4% (n=154) were transferred from primary facilities. More than 41.9% (n=164) arrived over one hour after injury.

Radiographs were used in 51.4% (n=201), CT scans in 19.7% (n=77), and 3.1% (n=13) received both X-ray and ultrasound; 12% (n=47) had no documentation.

Among survivors (93.5%, n=359), 60.4% (n=236) were discharged, and 31.5% (n=123) were admitted, mostly to general wards (86%, n=107), and some to ICU (14%, n=16). Twenty-five patients (6.4%) died.

## Discussion

This study provides insight into the nature, severity, and outcomes of RTIs in southwestern Ethiopia. Consistent with other studies (12–15), males were disproportionately affected, likely due to increased outdoor activity, occupational exposure, and driving responsibilities (12,16).

Victims under 20 years were most affected, aligning with national patterns but contrasting studies from India and Bangladesh (17,18). This reflects Ethiopia's youthful population and highlights their vulnerability.

Pedestrians were the most common victims (52.9%), consistent with other Ethiopian and Iranian studies (15,19), though differing from reports from Hawasa and Adama, where passengers were more commonly injured. The high pedestrian injury rate may result from poor road safety knowledge and infrastructure (9,20).

Musculoskeletal and head injuries were the most common. Although past studies from Ethiopia identified head trauma as predominant (15,23,24), our findings align more with data from Bangladesh, India, and Tanzania (18,21,22), perhaps due to the high number of pedestrian injuries.

Ambulance use (44.5%) was notably higher than in Yemen, India, and other Ethiopian regions (16,26–28), suggesting improved emergency response infrastructure in Jimma. Early ambulance access correlates with reduced mortality and better outcomes (29).

Severe injuries (62.2%) were more frequent compared to previous studies in Uganda and Ethiopia (15,30), yet the mortality rate (6.4%) was relatively lower, possibly due to better prehospital care and early treatment access.

This study did not assess long-term outcomes. Some patients might have died post-discharge. Incomplete records also limited analysis of certain variables.

In conclusion, the burden of RTIs is substantial, with young males and pedestrians being the most affected. Common injuries include musculoskeletal and head trauma. First aid at the scene was often lacking, and medical records were frequently incomplete. Addressing these gaps through targeted awareness, healthcare worker training, improved emergency services, and robust data systems is essential. Establishing a multi-sectoral injury surveillance system is crucial for identifying risk factors and designing effective prevention strategies.

## Figures and Tables

**Figure 1:**
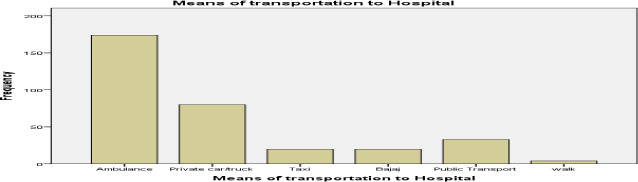
Means of transportation of victims of Road Traffic Accident to Jimma Medical Center, Jimma, Ethiopia, September–June 2021/22

**Figure 2:**
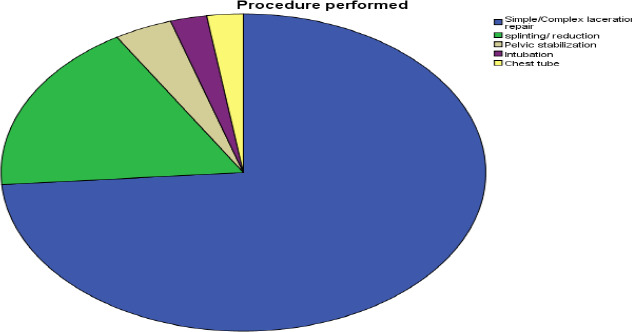
Procedure performed for victims of Road Traffic Accident at Jimma Medical Center, Jimma, Ethiopia, September–June 2021/22
